# Dataset for transcriptome and physiological response of mature tomato seed tissues to light and heat during fruit ripening

**DOI:** 10.1016/j.dib.2020.106671

**Published:** 2020-12-24

**Authors:** Elise Bizouerne, Benoit Ly Vu, Joseph Ly Vu, Jerome Verdier, Julia Buitink, Olivier Leprince

**Affiliations:** Institut Agro, Univ Angers, INRAE, IRHS, SFR 4207 QuaSaV, 49000 Angers, France

**Keywords:** Embryo, Endosperm, Heat, Fruit ripening, Light, Seed development, Tomato

## Abstract

Seed vigor is an estimate of how successfully a seed lot will establish seedlings under a wide range of environmental conditions, with both the embryo and the surrounding endosperm playing distinct roles in the germination behaviour. Germination and seedling establishment are essential for crop production to be both sustainable and profitable. Seed vigor traits are sequentially acquired during development via genetic programs that are poorly understood, but known to be under the strong influence of environmental conditions. To investigate how light and temperature have an impact on the molecular mechanisms governing seed vigor at harvest, RNA sequencing was performed on *Solanum lycopersicum* cv. Moneymaker seed tissues (i.e. embryo and endosperm) that were dissected from fruits that were submitted to standard or high temperature and/or standard or dim light. The dataset encompassed a total of 26.5 Gb raw data from mature embryo and endosperm tissues transcriptomes. The raw and mapped reads data on build SL4.0 and annotation ITAG4.0 are available under accession GSE158641 at NCBI Gene Expression Omnibus (GEO) database. Data on seed vigor characteristics are presented together with the differentially expressed gene transcripts. GO and Mapman annotations were generated on ITAG4.0 to analyse this dataset and are provided for datamining future datasets.

## Specifications Table

**Table:** 

Subject	Biological sciences
Specific subject area	Omics: Transcriptomics Plant Science: Plant Physiology
Type of data	Table Figure
How data were acquired	High-throughput RNA sequencing using BGISEQ-500
Data format	Raw reads (FASTQ) filtered and analysed with statistical tests. Percentages and speed of germination.
Parameters for data collection	Total RNA was extracted from isolated embryo and endosperm tissues of mature *Solanum lycopersicum* (cv Money Maker) seeds that were harvested at 70 days after flowering (DAF) from fruits that were ripened *ex planta* from the breaker stage (63 DAF) onwards under standard temperature (23 °C day/ 20 °C night) or high temperature (32 °C/26 °C) and under standard light regime (16 h photoperiod, 300 µE m^2^ s^−1^) or dim light (16 h photoperiod, 25 µE m^2^ s^−1^). Seed vigor was assessed during imbibition at 20 °C in the dark in water, 71 mM NaCl or a polyethylene glycol solution (PEG 8000) both corresponding to −0.3 MPa.
Description of data collection	RNA sequencing of total RNA followed by mapping and bioinformatic analysis for differential gene expression and gene set enrichment analyses. Germination data.
Data source location	Institution: Institut de Recherche en Horticulture et Semences, INRA City: Beaucouzé Country: France
Data accessibility	Repository name: NCBI GEO Data identification number: GSE158641 Direct URL to data: https://www.ncbi.nlm.nih.gov/geo/query/acc.cgi?acc=GSE158641https://data.mendeley.com/datasets/6h44fvz8×9/1

## Value of the Data

• This is a tissue-specific seed transcriptome dataset for tomato obtained from fruits that were ripened ex planta under four environmental conditions (high/standard temperature and standard/low light intensity).
• These data are a useful resource for the scientific community studying the developmental programs of various seed tissues and working on the effect of maternal environments on seed vigor. Annotation files of Gene Ontology or Mapman format are provided for the recently published SL4.0 genome version and can be used for enrichment analysis and data mining.
• These data provide new insights on tissue-specific molecular processes affected by heat and light leading to defects in seed vigor. They allow the identification of candidate genes as well as molecular markers that might predict seed vigor on tomato.
• The dissection of seeds into embryo and endosperm will contribute to decipher what the underlying molecular events are in the different tissues that determine seed vigor.

## 1 Data Description

**Fig. 1:**
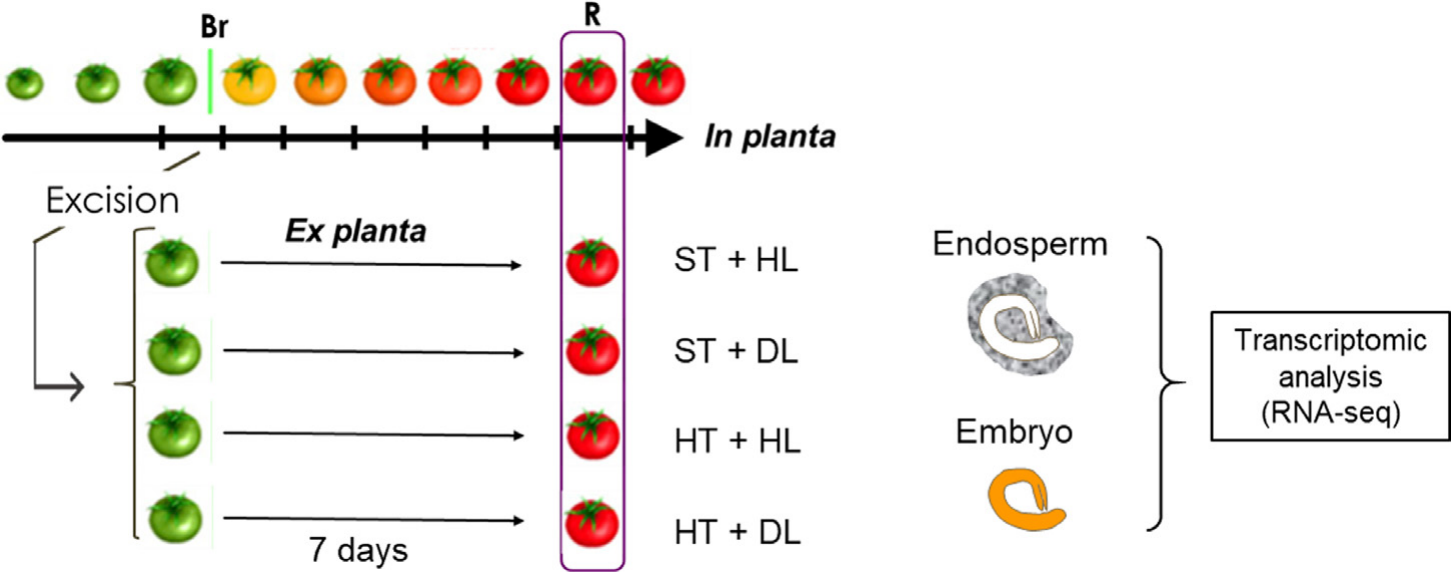
Experimental design. Fruits at breaker stage (i.e 63 DAF) were collected from plants grown in the greenhouse and transferred to growth chambers with 4 different environments: After 7 days, seeds were collected for physiological tests and seed tissues were dissected for RNA-sequencing. HT, high temperature; Br, breaker; DAF, days after flowering; DL, dim light; HL = high light; R, red. (For interpretation of the references to color in this figure legend, the reader is referred to the web version of this article.)

**Fig. 2:**
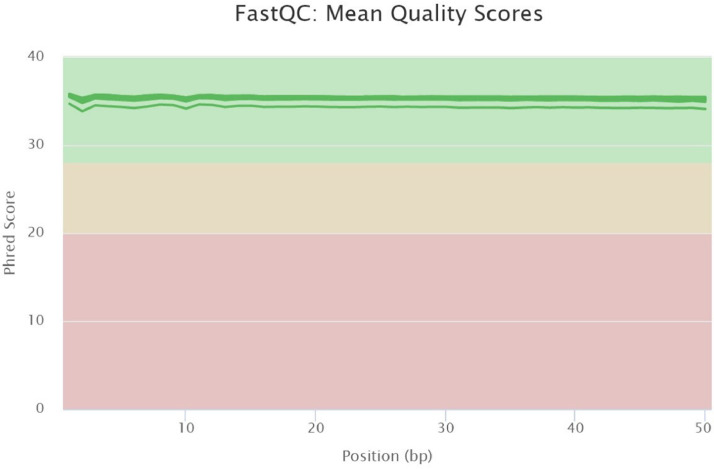
Overview of the range of quality values across all bases at each position in the fastq files obtained from FastQC.

**Fig. 3:**
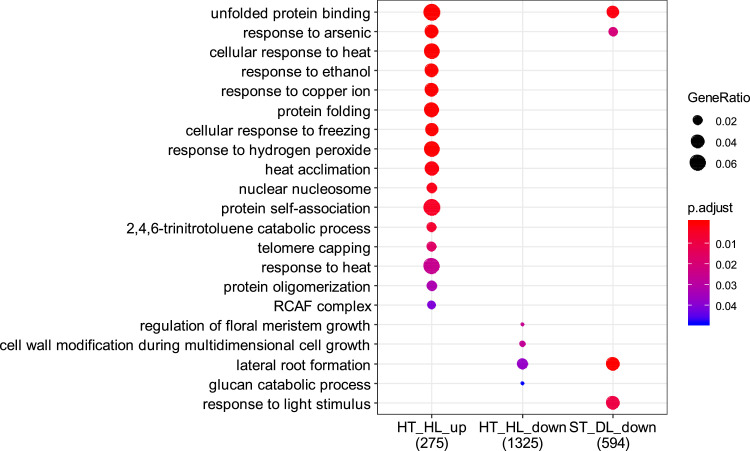
Major overrepresented biological functions of DEGs in in the embryo of seeds from fruits ripened under different light/temperature conditions compared to standard conditions. Enriched GO Terms were identified using ClusterProfiler algorithm with a hypergeometric test and a Bonferroni *p*-value adjustment threshold was set at 0.05 to control false positive rate. DL = dim light; HL = high light; HT = high temperature; ST = standard temperature.

**Fig. 4:**
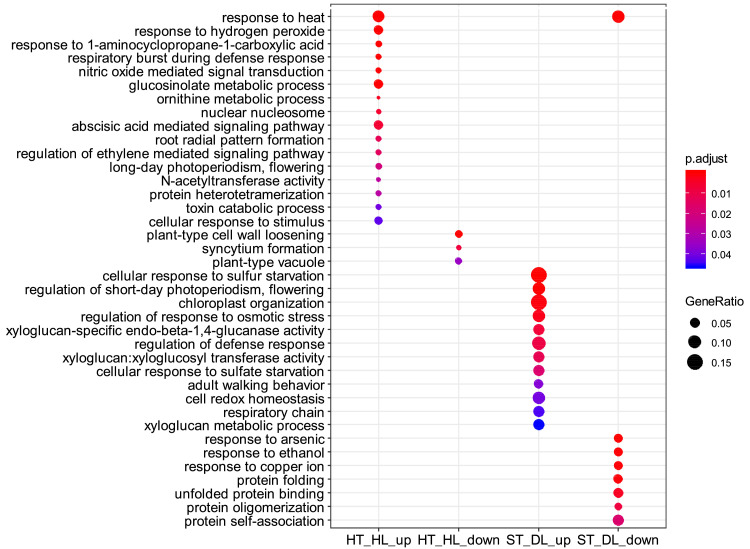
Major overrepresented biological functions of- DEGs in the endosperm of seeds from fruits ripened under different light/temperature conditions compared to standard conditions. Enriched GO Terms were identified using ClusterProfiler algorithm with a hypergeometric test and a Bonferroni *p*-value adjustment threshold set at 0.05 to control false positive rate. DL = dim light; HL = high light; HT = high temperature; ST = standard temperature.

This article presents a dataset of mRNA sequencing transcriptome profiling from isolated embryo and endosperm tissues of mature tomato (*Solanum lycopersicum* cv. Moneymaker) seeds that were isolated from fruits that were ripened *ex planta* from breaker stage onwards under standard or a combination of stressful conditions. Information on the experimental design of the study is shown in Fig. 1. The four environmental conditions were 1) standard temperature (ST) of 23 °C/20 °C and standard light, 300 µE m^2^ s^−1^, hereafter referred to as high light (HL), 2) ST and dim light (DL, 25 µE m^2^ s^−1^), 3) high temperature (32 °C/26 °C) and HL, and 4) HT and DL. The effects of the different fruit maturation environments on final seed vigor traits are shown in Table 1. Table 2 shows the quality of the transcriptome data and the mapped sequences on the reference tomato transcriptome build SL4.0 and annotation ITAG4.0 that is available at the Solgenomics website (ftp://ftp.solgenomics.net/tomato_genome/annotation/ITAG4.0_release/ [1]). On average 21 million reads out of the 25 million read sequenced per sample were mapped on the reference transcriptome (Table 2). Sequencing quality was checked using FastQC mean quality scores (Fig. 2). All samples displayed high quality scores with Phred scores around 35. The number of up- or down-regulated genes (DEGs) between standard maturation condition (standard temperature (ST) + high light (HL)) and stressful environments (ST + dim light (DL), high temperature (HT) + HL and HT + DL) are shown in Table 3. GO and Mapman annotations of ITAG4.0 were generated and are provided in Table S1 and Table S2, respectively. Enriched GO terms for up- and down- DEGs between standard ripening condition and stressful environments are shown in Fig. 3 for the embryo and Fig. 4 for the endosperm.

**Table 1:** Vigor characteristics of seeds extracted from fruits that ripened *ex planta* at different temperature and light conditions. After drying, seeds were imbibed in the dark at 20 °C in water, 71 mM Nacl or a PEG 8000 solution corresponding to −0.3 MPa. Data are the mean of three replicates of 50 seeds. Values in brackets represent standard deviation. DL = dim light; HL, high light; HT, high temperature; ST, standard temperature, t50, time of imbibition necessary to reach 50% germination).

Vigor trait	ST_HL	ST_DL	HT_HL	HT_DL
Germination H_2_O	98.0 (2.0)	86.0 (2.0)	78.7 (3.1)	86.0 (4.0)
Speed of germination (t50, d)	2.8 (0.1)	3.7 (0.1)	3.0 (0.1)	2.7 (0.2)
Germination −0.3 MPa	31.3 (8.1)	6.7 (4.2)	1.3 (1.2)	3.3 (2.3)
Germination 71 mM NaCl	23.3 (7.0)	16.0 (2.0)	14.0 (5.3)	12.0 (5.3)

**Table 2:** Summary of mapping information performed with SALMON and FastQC. End, endosperm; Em, embryo, DL = dim light; HL, high light; HT, high temperature; ST, standard temperature.

Sample Name	% Aligned	M Aligned	% Dups	% GC	M Seqs
HT_DL_Em_1	82.00%	20.7	64.60%	42%	25.2
HT_DL_Em_2	83.60%	20.9	51.10%	43%	25.0
HT_DL_Em_3	84.80%	21.5	61.90%	43%	25.3
HT_DL_Em_4	83.60%	21.1	65.40%	43%	25.2
HT_DL_End_1	82.70%	20.7	64.60%	42%	25.0
HT_DL_End_2	82.30%	20.8	62.80%	42%	25.3
HT_DL_End_3	83.10%	21.0	62.60%	42%	25.3
HT_HL_Em_1	81.60%	20.5	62.30%	42%	25.2
HT_HL_Em_2	81.60%	20.7	60.50%	42%	25.4
HT_HL_Em_3	80.80%	20.4	62.80%	42%	25.2
HT_HL_End_1	82.90%	21.1	63.60%	42%	25.4
HT_HL_End_2	83.40%	21.0	64.00%	42%	25.2
HT_HL_End_3	82.50%	20.9	64.40%	43%	25.4
ST_DL_Em_1	83.40%	20.8	57.10%	43%	24.9
ST_DL_Em_2	82.70%	20.8	59.70%	42%	25.2
ST_DL_Em_3	83.30%	20.8	66.90%	42%	24.9
ST_DL_End_1	82.10%	20.7	64.40%	42%	25.3
ST_DL_End_2	82.70%	21.0	64.50%	42%	25.4
ST_DL_End_3	82.60%	21.0	64.50%	42%	25.4
ST_HL_Em_1	81.90%	20.6	53.20%	42%	25.1
ST_HL_Em_2	83.30%	21.1	60.70%	42%	25.4
ST_HL_Em_3	83.20%	20.8	51.70%	43%	25.0
ST_HL_Em_4	82.10%	20.8	62.80%	42%	25.3
ST_HL_End_1	82.30%	20.8	62.60%	42%	25.3
ST_HL_End_2	83.40%	21.1	63.20%	42%	25.3
ST_HL_End_3	82.60%	20.9	61.70%	42%	25.3

**Table 3:** Number of differentially expressed genes (DEGs) in embryo and endosperm tissues in different light/temperature conditions compared to standard conditions. Number of DEGs were identified using the DEseq2 algorithm with a Benjamini-Hochberg p-value adjustment threshold set at 0.05 to control false positive rate. DL = dim light; HL, high light; HT, high temperature; ST, standard temperature.

		HT_HL / ST_HL	ST_DL / ST_HL	HT_DL / ST_HL
Embryo	up	358	82	1
	down	1561	692	6
Endosperm	up	610	60	153
	down	657	253	113

## 2 Experimental Design, Materials and Methods

### 2.1 Plant material and growth conditions

Plants of *Solanum lycopersicum* cv. Moneymaker were grown under controlled greenhouse conditions in 10 L pots containing substrate (Irish peat, perlite, coconut fiber; 50/40/10; v/v/v), watered with a nutrient solution and supplemented with 16 h of 250 µmol m^−2^ s^−1^ light. The day and night temperatures were respectively maintained at 23 °C and 20 °C. Breaker fruits (i.e. 63 DAF) were collected from the 3rd to 6th trusses and transferred to a growth chamber for 7 days under 4 different environments: standard temperature (ST, 23 °C day/20 °C night) + high light (HL, 16 h photoperiod 300 µE m^−^^2^ s^−1^), ST + dim light (DL, 16h photoperiod 25 µE m^−^^2^ s^−1^), high temperature (HT, 32 °C day/26 °C night) + HL or HT + DL. For seed vigor analyses, seeds were collected and incubated for 1 h in 0.4 g/L pectolytic enzyme solution (Lafazym CL®, Laffort, France) followed by extensive washing with water. Then, seeds were blotted dry on filter paper and rapidly dried at 43% RH under airflow at room temperature for 2d and stored in hermetically sealed bags at 4 °C prior to seed vigor tests. For RNA extraction, three replicates of 10 seeds were collected from the equatorial section of 2 different fruits for each replicate. Embryo and endosperm were hand-dissected, then immediately frozen in liquid nitrogen and stored at −80 °C.

### 2.2 Seed vigor tests

To assess final percentage of germination, triplicates of 50 dried seeds were imbibed on filter paper (Whatman No1) in 9 cm diameter Petri dishes at 20 °C in the dark, either in water for 8 days, in −0.3 MPa polyethylene glycol (PEG 8000, Sigma) solution or in 71 mM NaCl solution (equivalent to −0.3 MPa) for 15 days. Seeds were considered germinated when the radicle had protruded 1 mm from the seed coat. Germination speed in water was determined by daily scoring of germinated seeds and calculated as the time for the seed lot to reach 50% of germination (t50) using the fit of a three-parameter log-logistic model.

### 2.3 RNA extraction, library construction and sequencing

Total RNA was extracted using the NucleoSpin® RNA Plant and Fungi kit (Macherey-Nagel, Germany), according to the manufacturer instructions (protocol 5.1, sample type “alfalfa seed” for embryo and “potato tuber” for endosperm) without the 56 °C incubation step. RNA samples were quality checked using a nanodrop spectrophotometer ND-1000 (NanoDrop Technologies) and a 2100 Bioanalyzer (Agilent Technologies, Santa Clara, CA, USA) (OD260/280 > 2.00, OD260/230 > 2.20, RIN>6.5, 28S/18S<1.0, baseline smooth). Samples were sent to Beijing Genomics Institute (https://www.bgi.com), Hong Kong, for library preparation and sequencing on BGISEQ-500 platform, generating an average 20M reads of 50bp per sample.

### 2.4 RNA analysis and functional annotation

After quality control of fastq files using FastQC [2], high-quality reads were mapped onto the reference tomato transcriptome build SL4.0 [1] and transcript abundances were quantified with Salmon algorithm (version 0.14.1) [3] using the quasi-mapping mode and the ‘–validateMappings’ and ‘–seqBias’ options. Before mapping, the reference genome was indexed with Salmon using k-mers of length 31. Coverage estimates and statistics of the reads mapping are presented in Table 2. Differential expression of transcripts were calculated via DESeq2 [4]. Transcripts were considered differentially expressed if log_2_ fold change (FC) was above 1 or below −1 and if Benjamini-Hochberg adjusted *p*-value threshold was below 0.05. Data on total counts and differential gene expression can be found at https://data.mendeley.com/datasets/6h44fvz8x9/1. Gene Set Enrichment Analysis (GSEA) on GO Terms were performed with hypergeometric test using clusterProfiler package (v3.10.1) in R [5]. GO Terms were considered as enriched if Bonferroni adjusted p-value threshold was below 0.05. Gene Ontology (GO) annotation on SL4.0 was generated using OmicsBox (https://www.biobam.com/omicsbox/, [6]) (Table S1) and Mapman annotation was generated using Mercator v4 [7] (Table S2). The total gene enrichment analysis can be found at https://data.mendeley.com/datasets/6h44fvz8x9/1.

## CRediT Author Statement

**Elise Bizouerne:** Investigation, Data curation, Visualization, Writing- Original draft preparation **Benoit Ly Vu:** Investigation **Joseph Ly Vu:** Investigation **Julia Buitink:** Conceptualization, Methodology, Writing- Reviewing and Editing **Jerome Verdier:** Methodology **Olivier Leprince:** Supervision, Conceptualization, Writing- Reviewing and Editing

## Declaration of Competing Interest

The authors declare that they have no known competing financial interests or personal relationships which have or could be perceived to have influenced the work reported in this article.

## Appendix Supplementary materials

Image, application 1

Image, application 2
